# Health and Human Rights Education in U.S. Schools of Medicine and Public Health: Current Status and Future Challenges

**DOI:** 10.1371/journal.pone.0004916

**Published:** 2009-03-18

**Authors:** L. Emily Cotter, Jonathan Chevrier, Wael Noor El-Nachef, Rohan Radhakrishna, Lisa Rahangdale, Sheri D. Weiser, Vincent Iacopino

**Affiliations:** 1 School of Public Health, University of California, Berkeley, California, United States of America; 2 The George Washington University School of Medicine and Health Sciences, Washington, D. C., United States of America; 3 Feinberg School of Medicine, Northwestern University, Chicago, Illinois, United States of America; 4 Joint Medical Program, Division of Health and Medical Sciences, University of California San Francisco and University of California, Berkeley, California, United States of America; 5 Department of Obstetrics and Gynecology, Stanford University Medical Center, Palo Alto, California, United States of America; 6 Positive Health Program, San Francisco General Hospital, University of California San Francisco, San Francisco, California, United States of America; 7 Center for AIDS Prevention Studies, University of California San Francisco, San Francisco, California, United States of America; 8 Human Rights Center, University of California, Berkeley, California, United States of America; 9 Department of Internal Medicine, University of Minnesota Medical School, Minneapolis, Minnesota, United States of America; CIET, Canada

## Abstract

**Background:**

Despite increasing recognition of the importance of human rights in the protection and promotion of health, formal human rights education has been lacking in schools of medicine and public health. Our objectives were: 1) to determine the nature and extent of health and human rights (HHR) education among schools of medicine (SOMs) and public health (SPHs); 2) to identify perceived barriers to implementing HHR curricula; 3) to learn about deans' interests and attitudes toward HHR education, and; 4) to identify factors associated with offering HHR education.

**Methods and Principal Findings:**

We conducted a cross-sectional survey among deans of all accredited allopathic SOMs and SPHs in the United States and Puerto Rico. Seventy-one percent of U.S. SOMs and SPHs responded. Thirty-seven percent of respondents indicated that their schools offered some form of HHR education. Main barriers to offering HHR education included competition for time, lack of qualified instructors and lack of funding. Among schools not offering HHR education, 35% of deans were interested in offering HHR education. Seventy-six percent of all deans believed that it was very important or important to offer HHR education. Multiple regression analysis revealed that deans' attitudes were the most important factor associated with offering any HHR education.

**Conclusion:**

Findings indicate that though a majority of deans of SOMs and SPHs believe that knowledge about human rights is important in health practice and support the inclusion of HHR studies in their schools, HHR education is lacking at most of their institutions. These results and the growing recognition of the critical interdependence between health and human rights indicate a need for SOMs and SPHs to work towards formal inclusion of HHR studies in their curricula, and that HHR competency requirements be considered to overcome barriers to its inclusion.

## Introduction

According to the Universal Declaration of Human Rights (UDHR), human rights serve as the foundation for freedom, justice and peace in the world [1]. Neglect or violations of these rights, whether civil, political, economic, social or cultural in character, may have profound effects on health [2]–[8]. Currently, nearly half of the world's people live on less than $U.S. 2.00 per day with their health and well-being jeopardized by food insecurity, unsafe drinking water, inadequate sanitation, and poor access to education and basic health services [9]. These conditions are amplified by war, forced migration, violence and torture, ecological instability, and denial of access to basic education and to the benefits of scientific progress [10], [11]. Social and economic policies that result in extreme global inequality eclipse the right to the basic necessities of human survival and may have profound effects on health status.

The conceptualization of human rights as essential conditions for health was first articulated in the mid-1990's [2], [3]; since then, health professionals have increasingly recognized the importance of human rights in the protection and promotion of individual and global health [4], [12]–[16]. The importance of human rights in medical and health practices has been recognized in a number of statements and publications by professional health organizations [17]–[20]. In 2000, the United Nations (UN) provided a detailed elaboration of state responsibilities to protect, promote and fulfill the right of individuals to the “highest attainable standard of physical and mental health” contained in Article 12 of the International Covenant on Economic, Social and Cultural Rights [21]. In its General Comment 14, the UN recognizes that health is a product of respect for many human rights, including “the rights to food, housing, work, education, human dignity, life, non-discrimination, equality, the prohibition against torture, privacy, access to information, and the freedoms of association, assembly and movement,” among others [22]. In 2002, the UN Commission on Human Rights appointed a Special Rapporteur on the Right to Health, who is charged with the duty of reporting on the status of the right to health around the world and making recommendations on appropriate measures to promote and protect this right [23]. In 2005, the UN Educational, Scientific and Cultural Organization (UNESCO) developed a Universal Declaration on Bioethics and Human Rights, which, among other goals, aims to promote respect for human dignity and protect human rights by ensuring “respect for the life of human beings, and fundamental freedoms, consistent with international human rights law” [24].

The importance of human rights as conditions for health is also evident from the UN Declaration of Commitment on HIV/AIDS, which acknowledges that the full realization of human rights is an essential element in all areas of the global response to the epidemic, and sets out specific goals and actions to realize those rights [25]. Together, health professionals and human rights advocates have developed a World Health Organization (WHO) Framework Convention on Tobacco Control, one of the most widely supported treaties in the history of the UN, to address the growing global epidemic of tobacco-related diseases [26]. In addition, physicians and other health practitioners and scientists who have applied their knowledge and skills for the protection and promotion of health and human rights (HHR) [27] have been awarded the Nobel Peace Prize [28], [29].

In 1999, The World Medical Association stated that medical ethics and human rights are an “integral part of the work and culture of the medical profession” and therefore the “teaching of Medical Ethics and Human Rights [should] be included as an obligatory course in their curricula” [30]. Despite the growing understanding of the significance of human rights in the protection and promotion of health and recognition of the need to teach human rights in health professional curricula, formal human rights education is lacking in schools of medicine (SOMs) and public health (SPHs). In 1996, a survey of all U.S. SOMs reported that approximately half of the schools included at least one of 16 human rights issues in their required bioethics courses [31]. The study may have overestimated the inclusion of human rights in medical school curricula as only 6 of the 16 issues pertained directly to human rights (10 pertained to bioethics) and the extent to which human rights and bioethics topics were addressed in the required bioethics courses was not assessed. It is important to distinguish bioethics from human rights: bioethical principles are codes of conduct that regulate clinical encounters with individual patients. They do not attempt to define health and well-being, nor do they indicate possible causes of human suffering [32], [33]. Human rights, however, encompass a broader concept that considers the social, economic, cultural, and political conditions that promote health and respect human dignity. In a survey of all 28 U.S. SPHs in 1996, five schools (17%) reported offering a HHR course [34]. In a 2002 assessment, 19% of American SPHs and 2% of SOMs were found to offer coursework in human rights [32].

The current status of HHR education and challenges to its inclusion in medical and public health curricula is not well known. We therefore conducted a cross-sectional survey among deans of all accredited allopathic medical and public health schools in the United States and Puerto Rico with the following aims: 1) to determine the nature and extent of HHR education among schools of medicine and public health; 2) to identify perceived barriers to implementing HHR curricula; 3) to learn about deans' interests and attitudes toward HHR education, and; 4) to identify factors associated with offering HHR education.

## Methods

Anonymous surveys were mailed to deans of the 125 accredited allopathic SOMs and 37 SPHs in the United States and Puerto Rico. Deans were given the option of designating a representative to complete and return the survey which could be returned via fax, post, or email.

Deans were contacted at least three times between June 2006 and May 2007 to request their completion of the survey, which was adapted from previous studies on HHR education [31], [32]. The following definition of “human rights” was used in the survey cover letter to deans:

Human Rights seek to promote the inherent dignity of all people without distinction of any kind and, according to the Universal Declaration of Human Rights, serve as the foundation for freedom, justice and peace in the world. In recent years, many have recognized human rights as essential conditions for health. These include civil and political rights (i.e. freedom from arbitrary deprivation of life, torture and ill treatment, freedom of thought, conscience, religion, opinion, expression, peaceful assembly, association, movement, and rights to equality before the law, due process and to participate in government, among others) which are interdependent and indivisible with economic, social and cultural rights (i.e. the right to work, fair wages, social security, basic education, and standards of living adequate for health and well-being, and freedom from hunger, among others).

Structured survey instruments were used to collect information on school location, size of the student body, HHR education status and format, and barriers to offering HHR education. Additionally, the survey explored deans' interest in developing future HHR education for students, their beliefs on the most appropriate format for educating students about HHR, their attitudes toward the importance of HHR education within their school, and attitudes toward the importance of health professionals' knowledge of HHR for use in their careers. Deans were requested to select one answer for most questions; however, they were allowed to check all options that applied when asked about the format of their current HHR curriculum, their beliefs of the most appropriate type of HHR curriculum, and barriers to HHR education. Out of 162 schools contacted, deans of 115 schools responded and seven deans of SOMs declined participation for a final sample size of 108 and a participation rate of 67% (SOM = 65%, 81/125; SPH = 73%, 27/37). Written informed consent was obtained prior to participation in the survey. This study was approved by the Committee for the Protection of Human Subjects at the University of California, Berkeley.

Since our study represents a large proportion of the target population of deans of all SOMs and SPHs, p-values for all statistical analyses are adjusted using a finite population correction. For count data, we applied the second-order correction developed by Rao and Scott [35], [36]. The corrected chi-squared (χ^2^) is then transformed into an *F* statistic by dividing χ^2^ by its degrees of freedom of *d_0_ = (R−1)(C−1)* where *R* is the number of rows and *C* is the number of columns. The *F* statistic is then taken to have numerator degrees of freedom equal to *d_0_* and denominator degrees of freedom equal to *(n−1)d_0_*. We also used multiple logistic regression models to identify factors associated with offering HHR education. The dependent variable was defined as offering any HHR education including an elective or required course or seminar, or one or more modules of an elective or required course. Independent variables included school type (SOM versus SPH), funding source (public versus private) and two variables assessing attitudes towards HHR education; these variables were determined based on responses to the following questions: 1) “How important do you feel it is to offer a human rights course or module in your Medical/Public Health curriculum?” and 2) “How important do you feel it is for students to understand the role of human rights in their future health practice?” Each variable was coded continuously as 0 = not at all important; 1 = somewhat important; 2 = important; and 3 = very important. Models were adjusted for school size (<200, 200–500, >500 students) and location as determined by U.S. census categories (Northeast, Midwest, South, West). A finite population correction equal to 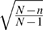 was also applied to coefficients obtained by multiple logistic regression.

Statistical analyses were performed using STATA version 10.0 (StataCorp, College Station, TX). Two-tailed finite population corrected p-values<0.05 were considered statistically significant.

References were identified through a search in PubMed using the key words health and human rights, with education, curriculum, global health, or bioethics.

## Results

### Survey Sample Characteristics

Forty-two percent of the deans identified their school as privately funded; 84% reported that at least 250 students were enrolled in their curriculum (Table S1). Our sample appeared to be representative of the nation's SOMs and SPHs as school type, funding source, location and student body size were similar in our sample compared with nationwide data (p>0.15 for all school characteristics).

### Nature and Extent of Health and Human Rights Education

Forty percent (42/105) of respondents reported that some form of HHR education (e.g., elective or required course or seminar, one or more module(s) of an elective or required course, or an elective conference) was offered at their institution (SOM = 32%, 25/79; SPH = 54%, 14/26) during the past academic year (Table S2). One or more required or elective HHR courses or seminars were reported to be offered by 22% of the institutions overall and were more than three times more prevalent among SPHs compared to SOMs (46% versus 14%; p<0.001). A significantly higher proportion of private schools offered such courses when compared to public schools (75% versus 33%, respectively; p<0.001).

### Barriers to Health and Human Rights Education

Among all respondents, the most frequently reported barrier to implementation of HHR courses was competition for time in students' schedules (82%). Other major barriers included lack of qualified instructors to teach the material (41%) and lack of funding (34%) (Table S3). Among deans of schools that did not offer HHR education, 12% of deans of SOMs cited lack of administrative support as a barrier while none of the deans of SPHs perceived this to be an obstacle (p<0.05). Additionally, among deans of schools not offering HHR education, those at public schools were significantly more likely than those at private schools to report lack of curriculum board support for HHR education (18% versus 5%, p<0.05) and more likely to perceive lack of student interest as a barrier (23% versus 5%, p<0.01).

### Interest in Health and Human Rights Education

Among schools not currently offering HHR education, 35% (22/62) of deans were interested in including it in their current curriculum. There were significantly more deans of SPHs compared to deans of SOMs who reported an interest in adding human rights coursework to their curricula (67%, 8/12, versus 28%, 14/50; p<0.01). Irrespective of their interest in offering HHR education, we asked all deans to identify formats that they believed were appropriate for HHR education in their curriculum: Fifty-eight percent thought that appropriate formats would include one or more modules of a required course, 45% a stand-alone elective course or seminar, and 32% one or more modules of an elective course. Deans of SPHs were more likely than deans of SOMs to believe that an elective course was appropriate for HHR education (70% versus 37%, p<0.01). Deans of private schools were more likely than those of public schools to believe that a module of a required course was appropriate for HHR training (75% versus 46%, p<0.01).

### Attitudes toward Health and Human Rights Education

Sixty-two percent of all respondents believed that it was very important or important to offer a HHR course or module, either required or elective (Table S4). Offering a HHR course or module to students was deemed more important by deans of SPHs compared to SOMs (p<0.01) and by deans of private schools relative to deans of public schools (p<0.001). Seventy-six percent of the deans reported that it was very important or important for students to understand the role of human rights in their future health practice. Deans of private schools were significantly more likely than deans of public schools to agree that it was important for students to understand the role of human rights in their future health practice (p<0.001).

### Factors Associated with Health and Human Rights Education

Schools headed by deans who had a more positive attitude towards offering HHR education were more likely to offer HHR courses in their curriculum. As shown in Table S5, this variable was the most strongly associated with HHR education (adjusted odds ratio (AOR) for each point increase on the attitudes scale = 4.3; 95% CI: 2.3, 7.8). Deans' attitudes regarding the importance of understanding human rights for future health practice were also strongly associated with HHR education in crude analyses but were no longer significant in multi-variable analyses (AOR = 1.2; 95% CI: 0.6, 2.4). In addition, schools that were privately funded had three times the odds of offering HHR education compared to schools that were publicly funded (AOR = 3.0; 95% CI: 1.4, 6.1), and SPHs were 2.6 times as likely to offer a HHR course (AOR = 2.6; 95% CI: 1.2, 5.5) compared to SOMs.

## Discussion

Approximately one-third of deans of SOMs and SPHs reported that some form of human rights education was offered at their institutions during the past year. The number of institutions offering required or elective HHR courses overall is considerably lower (22%). Approximately 66% of SOMs and 46% of SPHs do not currently offer HHR courses, and only 34% of all schools currently not offering HHR education are interested in incorporating it into their curricula in the future. HHR education may be even less common than these numbers suggest as deans of schools offering HHR education or interested in offering it may have been more likely to respond to our survey. Participants may also have over reported human rights education at their institutions. This is supported by the fact that the number of elective and required HHR courses in SPHs and SOMs reported in this study are approximately two and three times higher, respectively, compared to that reported in 2002 [32]. In addition, less than two-thirds of the elective and required HHR courses or seminars reported by the deans in this study are available in an updated listing of HHR courses offered by SOMs (5 courses) and SPHs (8 courses) reported by a consortium of health and human rights educators [37]. Although prospective participants received a detailed definition of HHR education, it is possible that they considered bioethics courses as human rights courses since both are concerned with respect for human dignity.

The most common barrier to HHR training reported by deans of SOMs and SPHs was competition for time in students' schedules. Other barriers identified were lack of qualified instructors, funding, administrative support and curriculum board support. The results of our multiple regression model suggest that lack of funding may be an important determinant of whether HHR training is offered as private schools were significantly more likely to offer HHR education compared with public schools. In addition, 42% of schools not offering HHR training and 50% of SPHs not offering HHR training reported funding as a barrier. These barriers can and should be addressed, as evidence suggests that there is strong student interest in HHR education. For example, students have initiated chapters of Physicians for Human Rights in nearly half (45%, 56/125) of all medical schools over the past 8 years, the majority of which were established in the past 2 years [38]. During the past year alone, students at 11 of these medical schools initiated HHR education electives, journal clubs and conferences independent of their curriculum [38]. In addition, 18 student groups have accessed online health and human rights course materials developed by the Human Rights Center at the University of California, Berkeley. A survey conducted in 46 countries, including the United States, demonstrated that medical students had a clear interest in human rights education; 55% of respondents believed a HHR framework should be compulsory, and even more believed that it was the duty of health professionals to be actively involved in the promotion of human rights [39]. In addition, other studies have shown that student interest in similar topics such as global health, health disparities, and vulnerable populations is at unprecedented levels [40], [41].

Deans of SOMs and SPHs reported favorable attitudes toward HHR education. Despite recognition of the importance of human rights in the promotion of health in medical and public health journals, by health professional organizations including the WHO and the UN, by deans of SOMs and SPHs, and by medical and public health students, there is no formal mandate for HHR education in professional health schools. While various barriers to HHR education exist, we found that deans' attitudes toward the importance of HHR training are the single most important factor associated with HHR education in U.S. SOMs and SPHs.

### Study Limitations

The findings of this study should be understood within the context of several limitations. Nearly 30% of deans of SOMs and SPHs did not respond to our multiple contact attempts. While our respondents seem representative of our target population, it is possible that deans of schools who did not offer HHR education or who held negative attitudes toward HHR education were less likely to respond. Moreover, although surveys were anonymous, deans may have felt pressured to report more positive attitudes toward HHR education out of social desirability, inflating the perceived importance of attitudes toward HHR education. In addition, surveys were only distributed to deans of allopathic SOMs and accredited SPHs; therefore, we do not know the status of HHR education or attitudes toward this education in other allied health professions, such as osteopathic medicine or nursing. Finally, because surveys were collected anonymously, it was not possible to determine how many responses came from deans or from their designated representatives.

### Interpretation

In the absence of a formal mandate and despite limited resources, HHR education has developed largely as a result of interest among students and human rights educators. To mitigate the apparent gap between deans' beliefs of the importance of human rights in health practice and the extent to which human rights are included in the education of physicians and public health practitioners, barriers to HHR education must be addressed. The disparity observed in the prevalence of HHR education based on funding source, combined with deans' beliefs that lack of funding is a barrier, indicates that funding is a significant factor related to the presence of HHR education in health professional schools. Other barriers to HHR education such as lack of qualified instructors and lack of faculty interest in teaching the material could be mitigated by HHR training for faculty and the development of HHR educational modules, which could be shared among educators at SOMs and SPHs. The content of basic HHR education modules should include core HHR concepts and a range of topics relevant to student and instructor interests. A review of HHR curriculum content in 2002 revealed that all of the courses included core concepts of international human rights law, health, and linkages thereof [32]. Additional topics most commonly included were: women's rights, health policy, war and refugees, bioethics, children's rights, torture, and economic, social and cultural rights [32]. Model HHR course materials are currently being developed by the Human Rights Center at the University of California, Berkeley with Internet access provided by Physicians for Human Rights in Cambridge, MA.

As deans' attitudes were significantly associated with the likelihood of HHR education being offered at their institution, it is possible that educating deans on the importance of HHR education may increase their interest in developing future HHR curricula. Raising awareness among deans and health educators may be facilitated by academic discourse and policy discussions within professional organizations such as the Association of American Medical Colleges and the Association of Schools of Public Health, and also by the development of HHR courses, modules and conferences at institutions where awareness is limited. Even in the absence of dean support, however, the establishment of HHR competency requirements appears to be warranted given the significance of the health and human rights framework for the promotion of global health and ethical health practices.

In the absence of the expressed duty to protect and promote human rights, health professionals have served as willing and unwilling accomplices in human rights violations, and with extraordinary health consequences [42]–[46]. The HHR discourse has developed to serve as a unifying framework to understand the role of health practitioners in society and provide practical tools for effective and socially relevant health policy and practice. As a non-partisan agenda for individual and global health, HHR education should be a critical component of the curriculum of SOMs and SPHs: it seeks not only to provide a foundation for the care of individuals who suffer illnesses, but to prevent the conditions that cause human suffering and to proactively promote the health and dignity of all people. Given the barriers to the inclusion of human rights in health education identified in this and other studies [32], the authors recommend a wide range of remedial measures to effectively integrate human rights into SOM and SPH curricula (Figure S1).

The findings of this study indicate that a majority of the deans of SOMs and SPHs believe that knowledge about human rights is important in health practice and that it is important to offer HHR studies in their schools. These findings and the growing recognition of the critical interdependence between health and human rights by students as well as national and international health organizations suggest the need for health professional schools to engage in formal inclusion of HHR studies.

## Supporting Information

Figure S1 (0.07 MB DOC)

Table S1 (0.09 MB DOC)

Table S2 (0.11 MB DOC)

Table S3 (0.12 MB DOC)

Table S4 (0.12 MB DOC)

Table S5 (0.05 MB DOC)
