# Supplementary material for: Health and Human Rights Education in U.S. Schools of Medicine and Public Health: Current Status and Future Challenges
Source: PLoS One. 2009 Mar 18;4(3):e4916. doi: 10.1371/journal.pone.0004916 (PMC2654657; doi:10.1371/journal.pone.0004916)
Supplement: Figure S1 — (0.07 MB DOC) [file pone.0004916.s001.doc]

**Figure S1.** Recommendations for the integration of Health and Human Rights education into SOM and SPH curricula.

- Foster leadership and support among SOM and SPH deans and health educators
- Establish HHR competency requirements for SOMs and SPHs
- Enhance discourse on practical applications of HHR in the health sector, e.g., special publication formats in academic and professional journals, HHR conferences and policy meetings, etc.
- Develop model curricular materials for courses and modules for graduate and continuing education, including Internet-based learning formats
- Implement HHR courses (required and elective) and modular HHR material in relevant courses, e.g., bioethics, global and international health, community medicine, health policy, etc.
- Train HHR instructors using on-site, conference, and Internet-based formats
- Establish and fund HHR fellowships and scholars programs and the expand existing post-graduate fellowship and scholars programs (e.g., Robert Wood Johnson, Rockefeller, Carnegie Mellon, Hastings Center, the Center for Disease Control, American Public Health Association, etc.) to include HHR topics
- Develop adequate financial support from public and private sources aided by the active support of deans and health educators and a formal mandate for HHR competencies
- Establish HHR centers at SOM and SPH institutions to coordinate and implement academic education, research activities, and discourse on HHR policies and practices
- Establish and further develop HHR divisions within professional health organizations including the American Medical Association, American Public Health Association, the Center for Disease Control, and the World Medical Association and the World Health Association
- Formally recognize the protection and promotion of human rights as professional duties of health practitioners
- Include human rights education in primary, secondary and undergraduate education to establish a foundation for critical understandings of human rights and for the development of public constituencies for human rights
