# Supplementary material for: Health and Human Rights Education in U.S. Schools of Medicine and Public Health: Current Status and Future Challenges
Source: PLoS One. 2009 Mar 18;4(3):e4916. doi: 10.1371/journal.pone.0004916 (PMC2654657; doi:10.1371/journal.pone.0004916)
Supplement: Table S1 — (0.09 MB DOC) [file pone.0004916.s002.doc]

**Table S1.** Characteristics of responding schools compared with national data.

| **Schools Characteristics** | **Study Sample (N = 108)^a^** | **National Data (N = 162)** |
| --- | --- | --- |
|  | **No. (%)** | **No. (%)** |
| Type of School |  |  |
| Public Health | 27 (25.0) | 37 (22.8) |
| Medicine | 81 (75.0) | 125 (77.2) |
|  |  |  |
| Funding Source |  |  |
| Private | 44 (41.9) | 60 (37.0) |
| Public | 61 (58.1) | 102 (63.0) |
|  |  |  |
| Number of Students |  |  |
| < 250 | 17 (15.9) | 13 (8.0) |
| 250 - 500 | 38 (35.5) | 67 (28.4) |
| > 500 | 52 (48.6) | 82 (50.6) |
|  |  |  |
| Geographic Location^b^ |  |  |
| Northeast | 23 (21.7) | 39 (24.1) |
| Midwest | 25 (23.6) | 37 (22.8) |
| South | 45 (42.5) | 64 (39.5) |
| West | 13 (12.3) | 22 (13.6) |

^a^Numbers may not add to 108 (total N) due to missing data. Percentages may not add to 100% due to rounding.

^b^Geographic location based on U.S. census categories:

Northeast (CT, ME, MA, NH, RI, VT, NJ, NY, PA)

Midwest (IL, IN, MI, OH, WI, IA, KS, MN, MO, NE, ND, SD)

South (DE, DC, FL, GA, MD, NC, SC, WV, AL, KY, MS, TN, AR, LA, OK, TX, VA; incl. PR)

West (AZ, CO, ID, MT, NV, NM, UT, WY, AK, CA, HI, OR, WA)
