# Supplementary material for: Health and Human Rights Education in U.S. Schools of Medicine and Public Health: Current Status and Future Challenges
Source: PLoS One. 2009 Mar 18;4(3):e4916. doi: 10.1371/journal.pone.0004916 (PMC2654657; doi:10.1371/journal.pone.0004916)
Supplement: Table S2 — (0.11 MB DOC) [file pone.0004916.s003.doc]

**Table S2.** Human rights education currently offered.^a^

| **Current format of HHR education** | **All Schools (n=105)** | **School Type** | | **Funding Source** | |
| --- | --- | --- | --- | --- | --- |
|  |  | **Public Health (n=26)** | **Medicine (n=79)** | **Private (n=44)** | **Public (n=61)** |
|  | **No. (%)** | **No. (%)** | **No. (%)** | **No. (%)** | **No. (%)** |
| Schools offering HHR | 39 (37.1) | 14 (53.8) | 25 (31.6)** | 24 (54.5) | 15 (24.6)*** |
| Required or elective course or seminar | 23 (21.9) | 12 (46.2) | 11 (13.9)*** | 18 (40.9) | 5 (8.2)*** |
| Modules of a required or elective course | 24 (22.9) | 6 (23.1) | 18 (22.8) | 12 (27.3) | 12 (19.7) |
| Elective conference or symposium | 2 (1.9) | 0 (0.0) | 2 (2.5) | 1 (2.3) | 1 (1.6) |
| Don't know | 0 (0.0) | 0 (0.0) | 0 (0.0) | 0 (0.0) | 0 (0.0) |
|  |  |  |  |  |  |
| Schools not offering HHR | 64 (61.0) | 12 (46.2) | 52 (65.8)** | 19 (43.2) | 44 (72.1)*** |
| Don’t know | 2 (1.9) | 0 (0.0) | 2 (2.5) | 0 (0.0) | 2 (3.3)* |

^a^Survey questions were: “To the best of your knowledge, have specific human rights courses or modules been offered within your school’s curriculum during the current academic year” and “What format(s) has (have) been used to teach human rights in your Public Health/Medical curriculum during the current academic year? [Circle ALL that apply];” Percentages add to more than 100% because respondents could select more than one answer; Numbers may not add to the total sample size due to missing data.

*p < 0.05; **p < 0.01; *** p < 0.001 comparing values by school type or funding source using a finite population correction [35,36].
