# Supplementary material for: Health and Human Rights Education in U.S. Schools of Medicine and Public Health: Current Status and Future Challenges
Source: PLoS One. 2009 Mar 18;4(3):e4916. doi: 10.1371/journal.pone.0004916 (PMC2654657; doi:10.1371/journal.pone.0004916)
Supplement: Table S3 — (0.12 MB DOC) [file pone.0004916.s004.doc]

**Table S3.** Barriers to inclusion of health and human rights education in schools curricula.a

| **Barriers to inclusion of HHR education** | **All Schools (n=108)** | **Schools not offering HHR training** | | | | |
| --- | --- | --- | --- | --- | --- | --- |
|  |  | **School Type** | | **Funding Source** | | **Total (n=64)** |
|  |  | **Public Health (n=12)** | **Medicine (n=52)** | **Private (n=19)** | **Public (n=44)** |  |
|  | **No. (%)** | **No. (%)** | **No. (%)** | **No. (%)** | **No. (%)** | **No. (%)** |
| Competition for time in students’ schedules | 89 (82.4) | 9 (75.0) | 46 (88.5)* | 15 (79.0) | 39 (88.6) | 55 (85.9) |
| Lack of a qualified instructor | 44 (40.7) | 5 (41.7) | 28 (53.9) | 10 (52.6) | 23 (52.3) | 33 (51.6) |
| Lack of funding | 37 (34.3) | 6 (50.0) | 21 (40.4) | 9 (47.4) | 18 (40.9) | 27 (42.2) |
| Lack of faculty interest to teach material | 22 (20.4) | 3 (25.0) | 16 (30.8) | 5 (26.3) | 14 (31.8) | 19 (29.7) |
| Lack of student interest | 14 (13.0) | 1 (8.3) | 10 (19.2) | 1 (5.3) | 10 (22.7)** | 11 (17.2) |
| Lack of curriculum board support | 11 (10.2) | 3 (25.0) | 6 (11.5)* | 1 (5.3) | 8 (18.2)* | 9 (14.1) |
| Lack of administrative support | 8 (7.4) | 0 (0.0) | 6 (11.5)* | 2 (10.5) | 4 (9.1) | 6 (9.4) |
| Not appropriate for curriculum | 1 (0.9) | 0 (0.0) | 1 (1.9) | 0 (0.0) | 1 (2.3) | 1 (1.6) |
| Not relevant | 3 (2.8) | 0 (0.0) | 2 (3.9) | 0 (0.0) | 2 (4.6) | 2 (3.1) |
| No significant barriers | 9 (8.3) | 1 (8.3) | 1 (1.9) | 1 (5.3) | 1 (2.3) | 2 (3.1) |

^a^ Survey question was: “Please identify any barriers that you believe may preclude the inclusion of future human rights courses or modules in your Public Health/Medical curriculum. [Circle ALL that apply];” Percentages add to more than 100% because respondents could select more than one answer; Numbers may not add to the total sample size due to missing data.

*p < 0.05; **p < 0.01 comparing values by school type or funding source using a finite population correction [35,36].
