# Supplementary material for: Health and Human Rights Education in U.S. Schools of Medicine and Public Health: Current Status and Future Challenges
Source: PLoS One. 2009 Mar 18;4(3):e4916. doi: 10.1371/journal.pone.0004916 (PMC2654657; doi:10.1371/journal.pone.0004916)
Supplement: Table S4 — (0.12 MB DOC) [file pone.0004916.s005.doc]

**Table S4.** Deans’ attitudes about health and human rights education.^a^

|  | **All Schools (n=106)** | **School Type** | | **Funding Source** | |
| --- | --- | --- | --- | --- | --- |
|  |  | **Public Health (n=27)** | **Medicine (n=80)** | **Private (n=44)** | **Public (n=60)** |
|  | **No. (%)** | **No. (%)** | **No. (%)** | **No. (%)** | **No. (%)** |
| Importance to Offer HHR Courses^b,c^ |  |  |  |  |  |
| Very Important | 18 (17.3) | 6 (22.2) | 12 (15.6)* | 11 (25.0) | 6 (10.5)** |
| Important | 48 (46.2) | 16 (59.3) | 32 (41.6) | 22 (50.0) | 25 (43.9) |
| Somewhat Important | 36 (34.6) | 5 (18.5) | 31 (40.3) | 11 (25.0) | 24 (42.1) |
| Not important | 2 (1.9) | 0 (0.0) | 2 (2.6) | 0 (0.0) | 2 (3.5) |
|  |  |  |  |  |  |
| Importance to understand human rights^d,e^ |  |  |  |  |  |
| Very Important | 37 (34.9) | 9 (34.6) | 28 (35.0)** | 23 (53.5) | 12 (20.0)** |
| Important | 44 (41.5) | 15 (57.7) | 29 (36.3) | 14 (32.6) | 29 (48.3) |
| Somewhat Important | 23 (21.7) | 2 (7.7) | 21 (26.2) | 6 (14.0) | 17 (28.3) |
| Not important | 2 (1.9) | 0 (0.0) | 2 (2.5) | 0 (0.0) | 2 (3.3) |

^a^Numbers in the table may not add to Ns shown in row headings due to missing data. Percentages may not add to 100% due to rounding.

^b^Survey question was: “How important do you feel it is to offer a human rights course or module (required or elective) in your Public Health curriculum? [Circle ONE]”

^c^Comparison of Public Health versus Medical school: p<0.01; comparison of Private versus Public Funding source: p<0.001.

^d^Survey question was: “How important do you feel it is for students to understand the role of human rights in their future health practice? [Circle ONE]”

^e^Comparison of Public Health versus Medical school: p<0.001; comparison of Private versus Public Funding source: p<0.001.

*p < 0.01; **p < 0.001 comparing values by school type or funding source using a finite population correction [35,36].
