# Supplementary material for: Health and Human Rights Education in U.S. Schools of Medicine and Public Health: Current Status and Future Challenges
Source: PLoS One. 2009 Mar 18;4(3):e4916. doi: 10.1371/journal.pone.0004916 (PMC2654657; doi:10.1371/journal.pone.0004916)
Supplement: Table S5 — (0.05 MB DOC) [file pone.0004916.s006.doc]

**Table S5.** Factors associated with currently offering health and human rights education (n=97).

|  | **Crude OR (95% CI)** | **Adjusted OR (95% CI)^a^** |
| --- | --- | --- |
| Importance to offer HHR education^b^ | 4.6 (2.9, 7.3)** | 4.2 (2.0, 8.7)** |
| Importance to understand human rights^c^ | 3.2 (2.2, 4.7) | 1.2 (0.6, 2.4) |
| Funding source^c^ | 3.7 (2.2, 6.2)** | 3.0 (1.4, 6.1)* |
| School type^e^ | 2.4 (1.4, 4.2)* | 2.6 (1.2, 5.5)* |

^a^Models included all variables listed and were adjusted for school size and location.

^b^Survey question was: “How important do you feel it is to offer a human rights course or module (required or elective) in your Public Health/Medical curriculum?" Scores range from 1 (not at all important) to 4 (very important).

^c^Survey question was: “How important do you feel it is for students to understand the role of human rights in their future health practice?" Scores range from 0 (not at all important) to 3 (very important).

^d^Public (coded as 0) versus private (coded as 1) funding.

^e^School of Medicine (coded as 0) versus School of Public Health (coded as 1).

*p < 0.01; **p < 0.001 using a finite population correction [35,36].
